# Supernumerary kidney with pelvic communication and a single ureter

**DOI:** 10.1590/0100-3984.2016.0094

**Published:** 2018

**Authors:** Renata Mendes da Silva, Jorge Chaib Neto, Moaci Ferreira de Morais Júnior

**Affiliations:** 1 Hospital Universitário da Universidade Federal do Piauí (HU-UFPI), Teresina, PI, Brazil; 2 Universidade Federal do Piauí (UFPI), Teresina, PI, Brazil

**Figure 1:**
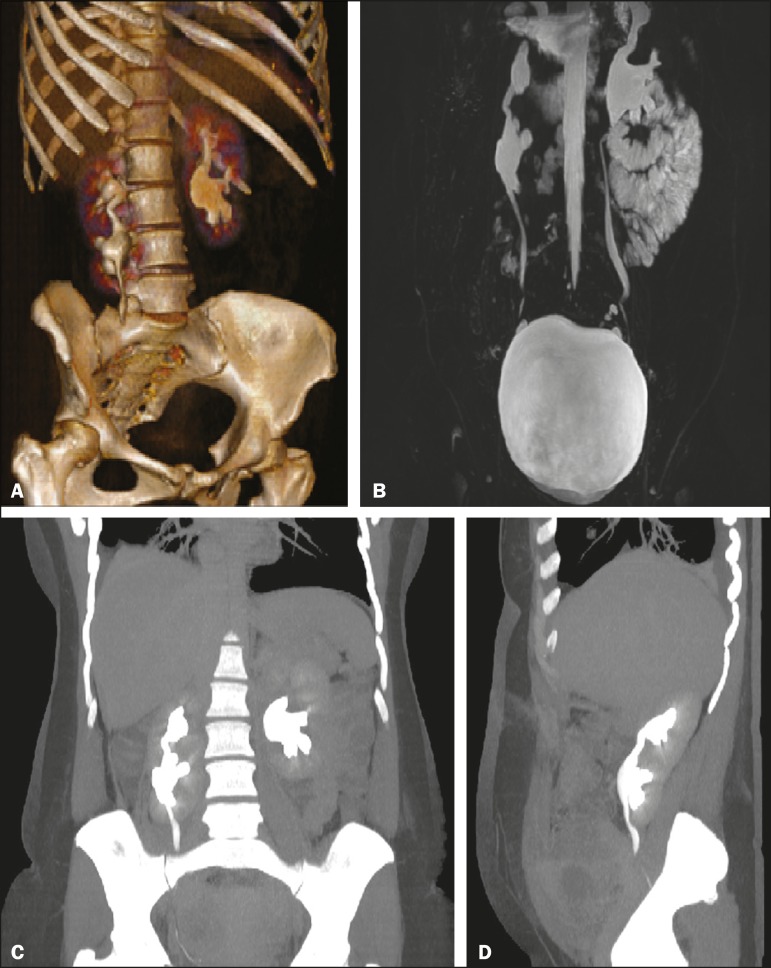
**A:** 3D reconstruction of a CT scan of the abdomen (excretory phase) showing a normal left kidney and a supernumerary kidney in a caudal position and anterior to the right kidney, with pelvic communication between them. **B:** T2-weighted fat-saturated enhanced fast gradient-recalled echo magnetic resonance imaging sequence (excretory phase), with 3D coronal reconstruction, showing pelvic communication between the normal right kidney and the supernumerary kidney, with a single ureter. **C,D:** Coronal and sagittal reconstructions of a CT scan of the abdomen (excretory phase), showing a normal left kidney, and a distinct and encapsulated reniform parenchymal mass, caudal and anterior to the right kidney, consistent with a supernumerary kidney, connected by a parenchymal bridge with pelvic communication between the two.

Dear Editor,

A 40-year-old female patient presented with diffuse abdominal pain after cholecystectomy with biliary tract exploration for choledocholithiasis and underwent computed tomography (CT) of the abdomen for better evaluation. The patient presented with no other comorbidities and was not taking any medications. The CT of the abdomen was performed with water-soluble iodinated contrast medium and three-dimensional (3D) reconstruction (Figures 1A, 1C, and 1D). In addition to liquid collections suggestive of biloma, CT revealed a distinct, encapsulated reniform parenchymal mass, anteriorly and near the lower pole of the right kidney, with a rotational anomaly. Subsequent magnetic resonance imaging of the urinary tract also demonstrated pelvic communication between the ipsilateral renal masses and a single ureter (Figure 1B).

Congenital anomalies of the urinary tract have been the object of recent studies in the radiology literature of Brazil^1-3^. A supernumerary kidney is a rare congenital anomaly of the urinary tract, fewer than 100 cases having been documented in the literature, with no difference between the genders and preferential occurrence on the left side. Because of its rarity, it typically goes undiagnosed until the fourth decade of life^4,5^. A supernumerary kidney has its own capsule, as well as its own blood supply, and can be totally separate from the ipsilateral kidney or attached to it by fibrous tissue or a parenchymal bridge. In general, the sum of the volume of ipsilateral fragments is equal to or greater than that of a normal kidney. The blood vessels that supply the supernumerary kidney typically originate from the aorta, and drainage is via the inferior vena cava^6,7^.

The embryological basis for the occurrence of a supernumerary kidney has not been fully elucidated. One of the main theories is that there is complete duplication of the ureteral bud, with independent penetration into the metanephric blastema, which develops and divides into two kidneys. Another theory is that there are two independent ureteral buds that penetrate the metanephric blastema, which then divides^7^. It is believed that a supernumerary kidney with a ureter that has its own insertion site in the bladder reflects an initial division of the mesenchyma before insertion and branching of the ureteral bud. A supernumerary kidney with a ureter that fuses with that of the normal kidney probably reflects late division of the metanephric mesenchyma^7^.

A supernumerary kidney can present as a palpable abdominal mass, with or without symptomatic nephrolithiasis, hydronephrosis, upper urinary tract infection, or renal tumors. However, it is typically asymptomatic and does not affect renal function. Therefore, they are never diagnosed or discovered incidentally^5^.
